# Genomic diversity and relationship analyses of endangered German Black Pied cattle (DSN) to 68 other taurine breeds based on whole-genome sequencing

**DOI:** 10.3389/fgene.2022.993959

**Published:** 2023-01-04

**Authors:** Guilherme B. Neumann, Paula Korkuć, Danny Arends, Manuel J. Wolf, Katharina May, Sven König, Gudrun A. Brockmann

**Affiliations:** ^1^ Animal Breeding Biology and Molecular Genetics, Albrecht Daniel Thaer-Institute for Agricultural and Horticultural Sciences, Humboldt-Universität zu Berlin, Berlin, Germany; ^2^ Department of Applied Sciences, Northumbria University, Newcastle Upon Tyne, United Kingdom; ^3^ Institute of Animal Breeding and Genetics, Justus-Liebig-Universität, Giessen, Germany

**Keywords:** *Bos taurus*, Holstein, whole-genome sequencing, inbreeding, phylogenetic analysis, runs of homozygosity, selection signatures, 1000 Bull Genomes project

## Abstract

German Black Pied cattle (Deutsches Schwarzbuntes Niederungsrind, DSN) are an endangered dual-purpose cattle breed originating from the North Sea region. The population comprises about 2,500 cattle and is considered one of the ancestral populations of the modern Holstein breed. The current study aimed at defining the breeds closest related to DSN cattle, characterizing their genomic diversity and inbreeding. In addition, the detection of selection signatures between DSN and Holstein was a goal. Relationship analyses using fixation index (F_ST_), phylogenetic, and admixture analyses were performed between DSN and 68 other breeds from the 1000 Bull Genomes Project. Nucleotide diversity, observed heterozygosity, and expected heterozygosity were calculated as metrics for genomic diversity. Inbreeding was measured as excess of homozygosity (F_Hom_) and genomic inbreeding (F_RoH_) through runs of homozygosity (RoHs). Region-wide F_ST_ and cross-population-extended haplotype homozygosity (XP-EHH) between DSN and Holstein were used to detect selection signatures between the two breeds, and RoH islands were used to detect selection signatures within DSN and Holstein. DSN showed a close genetic relationship with breeds from the Netherlands, Belgium, Northern Germany, and Scandinavia, such as Dutch Friesian Red, Dutch Improved Red, Belgian Red White Campine, Red White Dual Purpose, Modern Angler, Modern Danish Red, and Holstein. The nucleotide diversity in DSN (0.151%) was higher than in Holstein (0.147%) and other breeds, e.g., Norwegian Red (0.149%), Red White Dual Purpose (0.149%), Swedish Red (0.149%), Hereford (0.145%), Angus (0.143%), and Jersey (0.136%). The F_Hom_ and F_RoH_ values in DSN were among the lowest. Regions with high F_ST_ between DSN and Holstein, significant XP-EHH regions, and RoH islands detected in both breeds harbor candidate genes that were previously reported for milk, meat, fertility, production, and health traits, including one QTL detected in DSN for endoparasite infection resistance. The selection signatures between DSN and Holstein provide evidence of regions responsible for the dual-purpose properties of DSN and the milk type of Holstein. Despite the small population size, DSN has a high level of diversity and low inbreeding. F_ST_ supports its relatedness to breeds from the same geographic origin and provides information on potential gene pools that could be used to maintain diversity in DSN.

## 1 Introduction

Autochthonous populations are a crucial source of genetic diversity for the conservation of livestock species harboring important local adaptations (Medugorac et al., 2011). However, local breeds are typically less productive than intensively selected high-performing breeds. Consequently, keeping local breeds is less profitable (Gandini et al., 2007; Hiemstra et al., 2010). Thus, many local populations have been replaced by more profitable breeds which dramatically reduced the herd size of local populations. According to the Food and Agriculture Organization of the United Nations (FAO), 84% of all local breeds in Europe were considered at risk of extinction in 2021 (FAO, 2021).

This trend can also be observed for the German Black Pied cattle population (“Deutsches Schwarzbuntes Niederungsrind”, DSN). DSN is an endangered dual-purpose cattle breed from Germany (GEH e.V., 2020). Its initial farming dates back to the 18th century in the North Sea region of Germany and the Netherlands, where black and white animals were kept (today named DSN in Germany, and Dutch Friesian in the Netherlands). From there, black and white cattle were exported to North America and other parts of Europe. Strong selection on milk yield and dairy character resulted in the high-yielding dairy breed named Holstein Friesian (Holstein). These high-yielding cattle were brought back to Europe in the mid-1960s and rapidly became one of the main dairy cattle breeds worldwide. As a boomerang, DSN cattle were replaced almost entirely by Holstein (Brade, 2011; Brade and Brade, 2013). In 2020, the number of DSN herdbook cows was 2,452 (TGRDEU, 2021). According to the Society for the Conservation of Old and Endangered Livestock Breeds (GEH), populations with an effective population size (Ne) below 200 should be kept as genetic resources (Stier, 2021). For DSN, Ne was estimated as 85 (Jaeger et al., 2018). For that reason, this population became a genetic reserve in 1972, as a resource for the future of livestock breeding in Germany. This decision is in agreement with the Global Plan of Action for Animal Genetic Resources (FAO, 2007), in which it is stated that local breeds represent genetic resources that contain important alleles for the adaptation to local conditions (Hoffmann, 2011; Medugorac et al., 2011; Boettcher et al., 2014; Biscarini et al., 2015).

In this context, the breeding goal in DSN is to conserve the typical and beneficial characteristics of this breed, which are high robustness, fertility, longevity, resistance to multiple diseases, calm temperament, correct positioning of feet and legs, as well as high roughage feed intake capacity, making it suitable for grazing (BRS, 2021). Those characteristics confirm an advantage of DSN to organic farming. Not all those traits, however, are yet fully described in DSN and genetic variants affecting those traits are unknown. So far, studies on milk production (Korkuć et al., 2021), mastitis resistance (Meier et al., 2020), endoparasites infection resistance (May et al., 2019), and fertility traits (Wolf et al., 2021) identified some candidate variants and genes affecting those traits. To improve the identification of DSN-typical DNA variants underlying the phenotypic variance, a customized SNP chip was designed for DSN (Neumann et al., 2021). This DSN-specific SNP chip is currently used to support the characterization of genomic diversity and the identification of association between genetic variants and diverse phenotypes.

For an effective conservation plan, genomic diversity measurements are necessary to evaluate and ensure a minimum pool of genetic variants that provides sufficient adaptation capacity to changing environments and prevents inbreeding depression (Kristensen et al., 2015). Besides, typical measures of heterozygosity (observed and expected) and excess, of homozygosity, genome-wide nucleotide diversity, for example, has been shown to be useful when evaluating the genetic diversity status of a given population (Kardos et al., 2021). The nucleotide diversity is a region-wide metric that is used to quantify the degree of polymorphisms within a population. Additionally, runs of homozygosity (RoH) (McQuillan et al., 2008) have been used to detect inbreeding and signatures of selection on a genome-wide level (Mészáros et al., 2015; Mastrangelo et al., 2016; Gorssen et al., 2021).

Besides the joint historic origin of DSN and Holstein population (Naderi et al., 2020), little is known about which of the breeds that are maintained today are the most closely related to DSN. Due to the small population size of DSN, the risk of increasing inbreeding and losing diversity is high. Taking this into account, the breeds identified to be the most closely related to DSN on the genome level could potentially serve as genomic sources for maintaining and improving the genomic diversity within DSN and *vice versa*. If we look at the history and customs of people, we expect that breeds from the same region near the North Sea show a high level of genetic proximity (Brade and Brade, 2013; Felius et al., 2014). For that reason, analyses of the fixation index (F_ST_), phylogeny, and admixture could provide important information about the relationship between DSN and other populations from the same or different geographical locations.

Information on the genomic diversity of local breeds such as DSN and its relationship to other breeds can be taken into account in livestock breeding for maintaining the diversity within a breed and improving resilience. In the case of inbreeding depression or the spread of tropical diseases due to climate change, for example, known genomic regions improving disease resistance could be used for genetic rescue programs (Medugorac et al., 2011; Kristensen et al., 2015; Kardos et al., 2021). This would be especially useful for closely related breeds such as DSN and Holstein. Since DSN cattle are maintained under less selection intensity than Holstein, we expect that DSN cattle contain sequence variants that increase phenotypic plasticity and resilience.

The aim of the current work was to characterize the genomic diversity of DSN and define the breeds most closely related to DSN. To obtain a better understanding of the genomic diversity of DSN, we calculated and compared genomic measurements (observed heterozygosity, expected heterozygosity, excess of homozygosity, nucleotide diversity, and genomic inbreeding) within DSN and between breeds. For the analyses, we used whole-genome sequencing data of 302 DSN cattle together with the sequence variants of 68 other taurine breeds obtained from the 1000 Bull Genomes Project (Hayes and Daetwyler, 2019). In order to support the diversity analyses and the definition of the breeds most closely related to DSN, relationship analyses (F_ST_, phylogeny, and admixture) were performed between DSN and the other breeds. To detect regions of high differentiation between DSN and Holstein, we searched for signatures of selection within and between these two breeds.

## 2 Materials and methods

### 2.1 Genomic data

Sequence variants from whole-genome sequencing data of 302 DSN animals (Neumann et al., 2021) and 1,388 animals of additional 68 taurine breeds and one Auroch (*Bos primigenius*) from the 1000 Bull Genomes Project (Run 9) (Hayes and Daetwyler, 2019) were used in this study. Among the sequenced DSN cattle, there were 12 key ancestors of the last 44 to 20 years. Data pre-processing, sequence read mapping, variant discovery and recalibration for DSN have been described previously (Neumann et al., 2021). Basically, we followed the same pipeline guidelines as for the data from the 1000 Bull Genomes Project, using the *Bos taurus* genome version ARS-UCD1.2 as reference (Rosen et al., 2020). From the 1000 Bull Genomes Project data, only breeds with at least five animals with a minimum average read depth of 8-fold were used (Table 1). The maximum number of animals per breed was restricted to 30, whereas these 30 animals were randomly selected. Exceptions were made for Holstein, where 150 animals were selected (with 30 animals randomly selected per country), and Red and White Dual Purpose, where all 42 available animals were used due to pre-knowledge about their genetic proximity to DSN (Neumann et al., 2021). The sequence variants from the 302 DSN and the other breeds were merged using BCFtools v.1.9 (Narasimhan et al., 2016). A total of 79,019,242 biallelic autosomal variants (72,329,983 SNPs and 6,689,259 indels) occurring in the tranche 99% (from the Variant Recalibration performed by 1000 Bull Genomes Project) and with a call rate ≥ 0.95, were considered in our analyses (Supplementary Figure S1).

**TABLE 1:** Number of animals selected per breed and analyses in which they were included. R stands for ‘Relationship’ including phylogeny and F_ST_ (all breeds), A stands for ‘Admixture’ (20 breeds with highest F_ST_), and D for ‘Diversity’ (24 breeds with ≥ 25 animals).

Breed	n	Analyses	Breed	n	Analyses	Breed	n	Analyses
Abondance	9	R	Gelbvieh	30	R, D	Red and White Dual Purpose	42	R, A, D
Altai	20	R	German Red Angler	10	R, A	Ringamålako	8	R
Angus	30	R, D	Groningen White Headed	10	R	Romagnola	21	R
Angus Red	18	R	Guernsey	20	R	Rotes Höhenvieh	6	R
Aubrac	5	R	Hanwoo	20	R	Salers	18	R
Auroch	1	R	Hereford	30	R, D	Scottish Highland	7	R
Ayrshire Finnish	30	R, D	Holstein	150	R, A, D	Shorthorn	29	R, D
Belgian Red White Campine	10	R, A	Holstein Red	17	R, A	Simmental	30	R, D
Blonded Aquitaine	30	R, D	Jersey	30	R, D	Swedish Red	30	R, D
Brown Swiss	30	R, D	Kalmykian	10	R	Swedish Red Polled	6	R, A
Buryat	19	R	Kholmogory	30	R, A, D	Tarentaise	11	R
Buša	10	R	Limousin	30	R, D	Traditional Danish Red	9	R
Charolais	30	R, A, D	Maine Anjou	20	R	Tyrolean Grey	17	R
Chianina	15	R	Marchigiana	9	R	Ukrainian Grey	8	R
Deep Red Cattle	9	R, A	Menggu	10	R	Väneko	11	R
DSN	302	R, A, D	Modern Angler	20	R, A	Vorderwälder	13	R
Dutch Belted	11	R, A	Modern Danish Red	28	R, A, D	Wagyu	28	R, D
Dutch Friesian Red	11	R, A	Montbeliarde	30	R, D	Western Finncattle	15	R
Dutch Improved Red	9	R, A	Normande	30	R, D	West Vlaams Rood	11	R, A
Eastern Belgian Red White	7	R, A	Northern Finncattle	19	R	Yakut	30	R, D
Eastern Finncattle	15	R, A	Norwegian Red	29	R, A, D	Yanbian	10	R
Eastern Flanders White Red	12	R, A	Original Braunvieh	30	R, D	Yaroslavl	22	R
Fjäll	17	R	Podolian Serbia	10	R	Total 1,691		
Fleckvieh	30	R, D	Polish Red	7	R, A			

For the phylogenetic tree and admixture analyses, the available 79,019,242 sequence variants were pruned (--indep-pairwise) with PLINK v2 (Purcell et al., 2007) using a *r* ^2^ threshold of 0.6, a window size of 50 SNPs, and a step-size of 5 SNPs to 23,059,286 variants. The same parameters for pruning were also used for other cattle analyses based on WGS (Xia et al., 2021; Zhang et al., 2022), except for a higher, more conservative *r* ^2^ of 0.6, used at this point to keep variants in medium LD. All 68 breeds were used in the phylogenetic tree and in the F_ST_ analyses. Subsequently, the 20 breeds most closely related to DSN according to the estimated F_ST_ values were used in the admixture analysis.

Diversity and inbreeding measures were calculated for 24 breeds (Table 1) which had at least 25 animals. According to the FAO recommendation, 25 is the minimum sample count for a precise genetic diversity description of a population (FAO, 2011). For these 24 breeds, the initial 79,019,242 sequence variants were filtered down to 34,856,428 variants segregating among the 1,118 animals of those 24 breeds.

### 2.2 Relationship analyses

#### 2.2.1 Phylogenetic tree

To study the relationship between the available breeds and to detect the most closely related breeds to DSN, a genome-wide phylogenetic tree was built for all *Bos taurus* autosomes (BTA). Based on 23,059,286 pruned sequence variants, Manhattan distances between animals were calculated and the Unweighted Pair Group Method with Arithmetic mean (UPGMA) algorithm implemented in the biotite library v0.35.0 (Kunzmann and Hamacher, 2018) in Python was used for clustering. The phylogenetic tree was visualized using iTOL v6 (Letunic and Bork, 2019). Individuals of the same breed were collapsed to a branch labelled with the breed’s name. One auroch (*Bos primigenius*) was added as an outgroup in order to root the tree. Animals of a specific breed clustering outside the expected breed branch were removed. In the case of multiple clusters for a single breed, only the biggest cluster was kept. In addition, to detect migration events, a maximum likelihood tree allowing two migration events with bootstrap in blocks of 500 variants was built using TreeMix v1.13 (Pickrell and Pritchard, 2012). Number of migrations (m) was defined based on the Evanno method (Evanno et al., 2005) as implemented in the R package OptM v0.1.6 (Fitak, 2021). Different m from 2 to 6 were tested and a maximum Δm = 8.66 was estimated when m = 2 edges were selected.

#### 2.2.2 F_ST_ calculation

Pairwise F_ST_ values between DSN and the other 68 breeds were calculated using variants segregating in either DSN or the other breed from the initial dataset of 79,019,242 sequence variants. F_ST_ values were estimated based on Hudson’s method (Hudson et al., 1992) using the scikit-allel v1.3.1 library (Miles et al., 2020) in Python. As discussed by Bhatia et al. (Bhatia et al., 2013), Hudson’s method performs better when the sample size per breed varies largely and demands less computational power for big datasets.

#### 2.2.3 Admixture

The population structure analysis was done with the Admixture v1.3 software (Alexander et al., 2009). For this analysis, we used the segregating sequence variants of the pruned dataset (the same used for the phylogenetic analysis) of the 20 breeds most closely related to DSN according to their F_ST_ values. The number of animals for DSN and Holstein was reduced to 50, which were selected based on kinship. The 50 least related animals were selected based on a genomic relationship matrix (Yang et al., 2011) calculated with PLINK v2 (--make-rel) following a greedy approach starting with a randomly selected animal. Unsupervised analyses were performed for K (number of ancestral populations) ranging from 2 to 20 with 5-fold cross-validation (CV), whereof *K* = 4 was considered for interpretation, with the lowest CV error (CV error = 0.1448). *K* = 5, 6, and 7, also showed very low CV errors (Supplementary Figure S2). Those results were visualized with the CLUMPAK v1.1 software (Kopelman et al., 2015). Admixture between breeds was confirmed based with f3 statistics calculated on TreeMix v1.13 software using the *threepop* function over blocks of 10,000 variants.

### 2.3 Diversity analyses

#### 2.3.1 Genomic diversity

The genomic variation within breeds was assessed as nucleotide diversity (π) (Nei and Li, 1979), and as observed (H_o_) and expected (H_e_) heterozygosity. H_o_ and H_e_ were estimated using vcftools v0.1.15 (Danecek et al., 2011), calculated relative to the total number of variants among all 24 breeds (34,856,428). This was done in order to remove bias on the number of segregating variants per breed, and to allow the comparison between breeds. A chi-squared test was performed between H_o_ and H_e_ for each breed using the package statsmodels v0.13.5 (Seabold and Perktold, 2010) in Python. Nucleotide diversity was calculated per window of 10 kb (π_window_) using the library scikit-allel v1.3.1 (Miles et al., 2020) in Python. The nucleotide diversity per chromosome was calculated as the mean (π_ChrMean_) and median (π_ChrMedian_) of all π_window_ values of the respective chromosome, and total nucleotide diversity as the mean (π_TotMean_) and median (π_TotMedian_) of all π_window_ values across the whole genome. Due to the non-parametric nature of the π_window_ distributions, we also report median values in the Supplementary Materials (Supplementary Table S4). Since π_TotMean_ and π_TotMedian_ are highly correlated (Pearson correlation coefficient of 0.99), both values allow the same interpretation of the results. We preferred means over medians since means are widely used for nucleotide diversity in the literature, providing opportunities for comparisons. All π measures are shown as percentages, which means that final results were multiplied by 100.

#### 2.3.2 Genomic inbreeding

Genomic inbreeding was assessed using two estimators:

(1) Excess of homozygosity (F_Hom_) which is based on the method of moments (Li and Horvitz, 1953) was calculated as

$$F_{\mathrm{Hom}}=\frac{number\;of\;observed\;homozygous-number\;of\;expected\;homozygous\;}{total\;number\;of\;variants-number\;of\;expected\;homozygous},$$

and

(2) Inbreeding coefficient F_RoH_ which is based on RoHs was estimated using BCFtools v1.9 with an assumed recombination rate of 10^–8^ per base pair (1 cM/Mb). Inbreeding was calculated for the 24 breeds that were used for diversity analysis. Allele frequency was estimated using vcftools v0.1.15 and used as an input parameter for BCFtools v1.9. RoHs were separated into five groups with the minimal lengths of 50 kb, 100 kb, 1 Mb, 2 Mb, and 4 Mb (McQuillan et al., 2008; Zhang et al., 2015; Forutan et al., 2018; Bhati et al., 2020; Dixit et al., 2020). F_RoH_ was calculated for each group as

$$F_{RoH}=\sum L_{RoH}/{{}_{L}}_{genome},$$

where *L*_*RoH*_ is the length of a homozygous region and *L*_*genome*_ the length of the genome covered by SNPs (2,487,849,970 bp for our dataset).

### 2.4 Signatures of selection

#### 2.4.1 Region-wide F_ST_ between DSN and Holstein

F_ST_ values between DSN and Holstein were compared across the whole genome in windows of 10 kb. Only windows containing at least five sequence variants were considered. The top 0.01 percentile of the F_ST_ values were selected to point to potential differences in selection signatures of those breeds.

#### 2.4.2 Cross-population-extended haplotype homozygosity

The cross-population-extended haplotype homozygosity (XP-EHH) (Sabeti et al., 2007) was calculated between DSN and Holstein using the R package rehh v3.2.2 (Gautier et al., 2017). All segregating variants occurring in DSN or Holstein were considered. Variants were initially phased using Beagle v5.1 (Browning et al., 2018). Positive XP-EHH scores represent variants positively selected in DSN compared to Holstein, and negative scores correspond to variants positively selected in Holstein compared to DSN. *p*-values were corrected for multiple testing using Bonferroni and variants with a *p*-value<0.05 were considered as significant. The positions of neighboring significant variants were considered together as significant XP-EHH region for gene annotation.

#### 2.4.3 RoH islands

RoH islands were defined as regions with the highest frequency of SNPs inside RoHs among DSN or Holstein. Frequency of SNP inside RoHs were calculated as the number of animals in which a SNP was reported inside a RoH, divided by the total number of animals in the breed. The threshold to define islands was taken as the top 0.05 percentile of frequencies. Afterwards, the positions of neighboring SNPs satisfying the defined percentile threshold were used to form islands.

#### 2.4.4 Gene annotation

Regions with high difference in F_ST_ values between DSN and Holstein, significant XP-EHH regions, and RoH islands ± 250 kb flanking the start and end positions were scanned for protein-coding genes using the Ensembl database release 106 and for QTLs stored in the CattleQTLdb release 47 (Hu et al., 2022). The flanking 250 kb regions were included in our search since linkage groups and haplotype blocks can be quite large in DSN and reach, e.g., in the casein region on BTA 6, even 1 Mb (Korkuć et al., 2021). From the CattleQTLdb, QTLs and associations for “production”, “exterior”, “meat and carcass” (meat), “milk”, “health”, and “reproduction” (fertility) traits of a length <10 kb were considered. The references for the QTLs and associations were retrieved using PubMed IDs through the metapub v0.5.5 Python package. The complete list of publications is shown in Supplementary Tables S7–S9.

## 3 Results

### 3.1 Phylogenetic analysis

**FIGURE 1:**
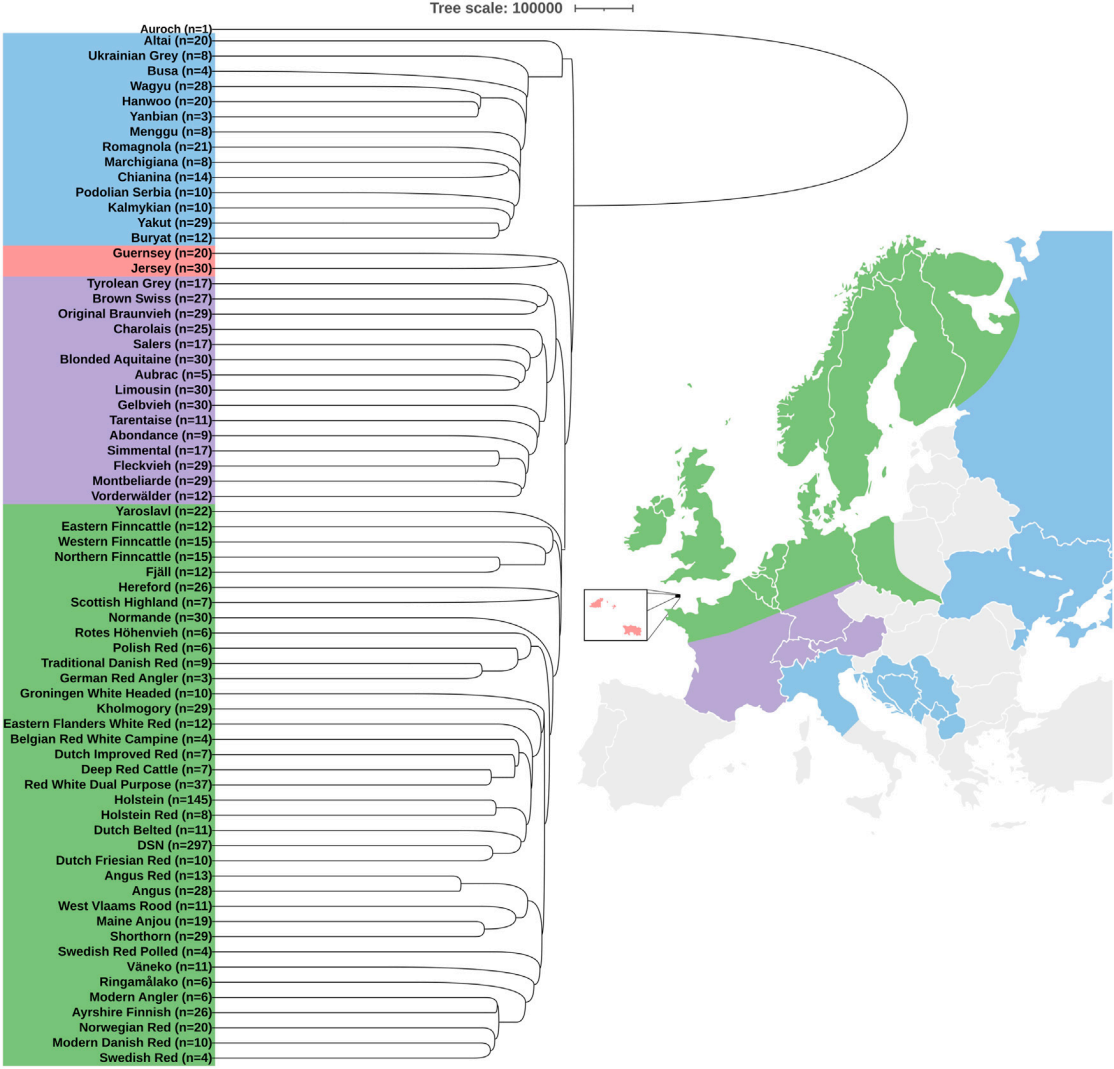
Phylogenetic tree and diversity analysis of 69 cattle breeds and Auroch as an outgroup. Colors in the phylogenetic tree represent geographical location of cattle origins: Northern Europe (green), Central Europe (violet), Jersey and Guernsey islands (red), and Eastern Europe together with Central Italy and Asia (blue). In parentheses, n is the number of animals representing the breed. Countries without any genomic breed information are highlighted in gray.

The phylogenetic analysis of DSN and 68 other cattle breeds including Auroch as an outgroup showed four clear clusters based on geographical origins (Figure 1). The majority of the breeds including DSN and Holstein formed a cluster of Northern European countries. Within this cluster, many Holstein Red cattle were found within the sub-cluster of Holstein cattle and *vice versa* (Supplementary Table S1), which was expected, since the coat color is the main difference. Also, animals of the breeds Modern Danish Red, Swedish Red, Norwegian Red, Ayrshire Finish, and Modern Angler were mixed with each other, showing wrong assignments or high relationship between those breeds exists (Supplementary Table S1). The cluster of Central Europe comprised all breeds from Austria, Switzerland, Southern Germany and France. Those are mainly dual-purpose breeds kept in mountainous areas. Jersey and Guernsey formed a separate cluster. The remaining cluster was formed by breeds from Eastern European countries, Central Italy, and Asian countries. This last cluster was the closest to Auroch.

We observed that DSN clustered closely to Dutch Friesian Red, Dutch Belted, Holstein, Holstein Red, Red White Dual Purpose, Deep Red Cattle, Dutch Improved Red, Belgian Red White Campine, Eastern Flanders White Red, Kholmogory, and Groningen White Headed. Those are all breeds originating from Germany, the Netherlands, and Belgium, except for Kholmogory which is from Russia, but crossbreeding with Friesian cattle was reported (Dmitriev and Ernst, 1987).

It is important to mention that the Finncattle breeds, Fjäll, Scottish Highland, Yaroslavl, and Altai clustered outside our reported clusters depending on the pruning parameters (Supplementary Figure S3). In addition, migration events were observed between an ancestor of Shorthorn and Maine Anjou to Charolais and from Traditional Danish Red to an ancestor of Polish Red and Rotes Höhenvieh (Supplementary Figure S4).

### 3.2 Relatedness to DSN using F_ST_

The same breeds as in the phylogenetic tree analysis showed the closest relationship to DSN in terms of lowest F_ST_ values. Those breeds with increasing F_ST_ values from 0.032 to 0.075 are Dutch Friesian Red, Dutch Improved Red, Eastern Belgian Red White, Belgian Red White Campine, Deep Red Cattle, Eastern Flanders White Red, Holstein Red, Kholmogory, Red and White Dual Purpose, Holstein, and Dutch Belted. Breeds which have also low F_ST_ values ranging between 0.053 and 0.078, but a little bit more distant in the phylogenetic analysis, are Modern Angler, Modern Danish Red, German Red Angler, Polish Red, Norwegian Red, Eastern Finncattle, West Vlaams Rood, and Swedish Red Polled (listed in increasing order). Charolais is the only breed that clustered outside the North Europe cluster in the phylogenetic tree, but had a low F_ST_ value of 0.070 to DSN. All F_ST_ values are listed in Supplementary Table S2.

### 3.3 Admixture

**FIGURE 2:**
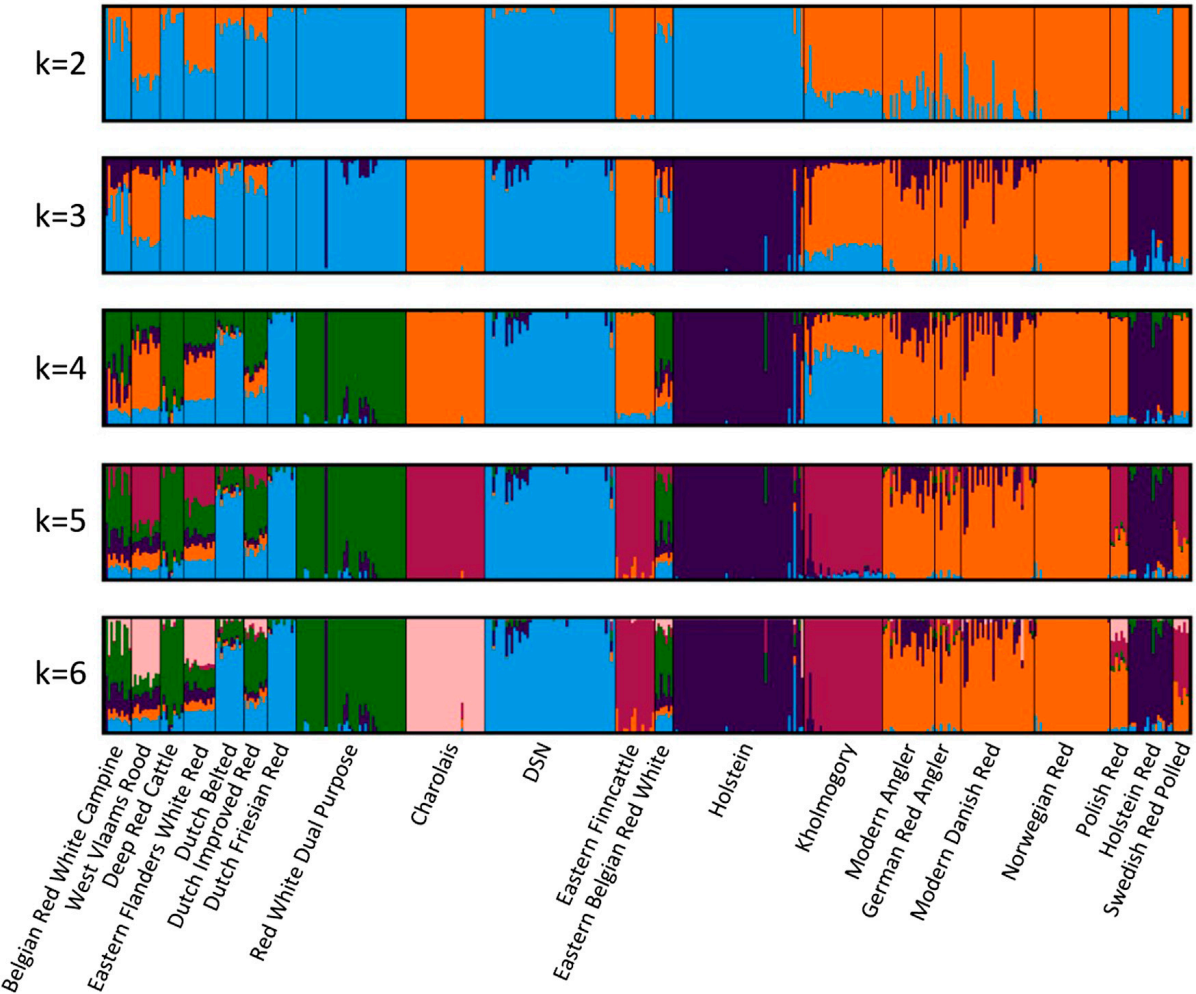
Admixture analysis between DSN and the 20 breeds closest to DSN according to the F_ST_ values. *K* = 4 was optimal for the 5-fold cross-validation. Colors indicate the ancestral relationship between breeds.

The admixture analysis (Figure 2) at *K* = 4 corroborated the results from the phylogenetic analysis by showing a common ancestral population between DSN, Dutch Friesian Red, Dutch Belted, Kholmogory, Dutch Improved Red, Eastern Flanders White Red, and Eastern Belgian Red White (in blue)—all breeds from the Netherlands and Belgium, except for Kholmogory. Polish Red, Swedish Red Polled, Modern Angler, Norwegian Red, German Red Angler, Modern Danish Red, Charolais, Eastern Finncattle, West Vlaams Rood, and Eastern Flanders White Red appear with a common ancestry (in orange). Holstein and Holstein Red share the same ancestry (in dark blue), with a clear level of introgression in most of the breeds, including DSN. Even though Charolais is the only breed located in a different cluster in the phylogenetic tree, admixture between Charolais, DSN, and breeds closely related to DSN exists. Admixture was detected by f3 statistics, whereof Charolais is significantly admixed from DSN and all the other 19 tested populations (Supplementary Table S3).

### 3.4 Genomic diversity

**FIGURE 3:**
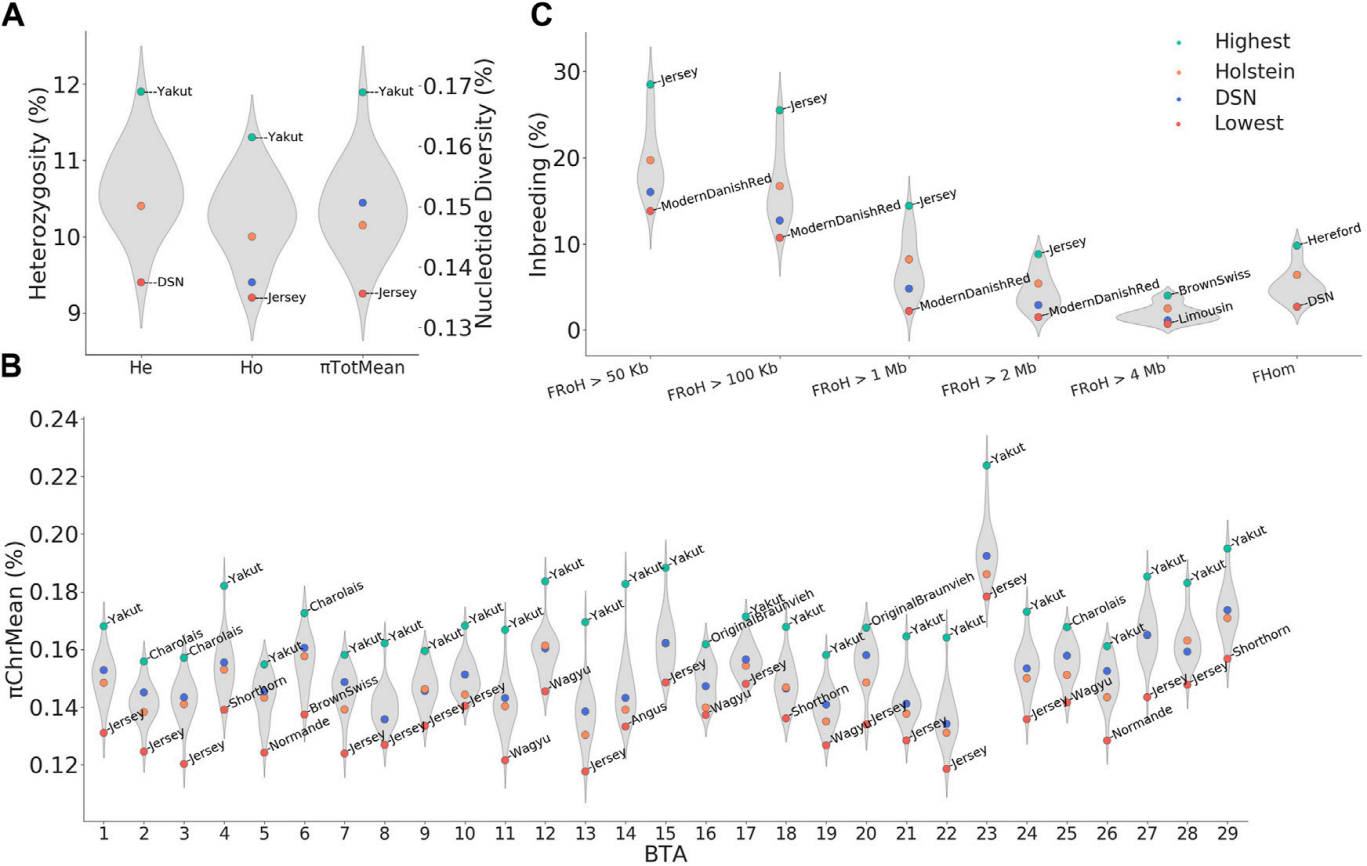
Overview of diversity measurements and inbreeding across breeds. **(A)** Average nucleotide diversity (π_TotMean_) observed heterozygosity (H_o_), andexpected heterozygosity (He) distributions, **(B)** average nucleotide diversity per chromosome (π_TotMean_), **(C)** inbreeding defined based on runs ofhomozygosity (F_RoH_) and excess of homozygosity (F_Hom_). Values for DSN and Holstein are highlighted in blue and orange, respectively. For each distribution, the highest and lowest values are highlighted in green and red, respectively, including the respective breed names.

The average value of the expected heterozygosity per individual (H_e_) ranged between 9.4% in DSN and 11.9% in Yakut and the average observed heterozygosity (H_o_) ranged between 9.2% in Jersey and 11.3% in Yakut (Figure 3A). Although the expected and observed heterozygosity did not significantly differ in DSN, absolutely, DSN did not have the lowest observed heterozygosity. With a few exceptions (Holstein, Norwegian Red, Blonded Aquitaine, Normande, Montbeliarde, Shorthorn, and Brown Swiss), most breeds did not show a H_o_ significantly lower than H_e_ (Supplementary Table S4).

The total average genomic diversity per individual π_TotMean_ ranged between 0.136% in Jersey and 0.169% in Yakut (Figure 3A). In DSN, π_TotMean_ was 0.151%. The DSN value was higher than in Holstein (0.147%) and in other breeds such as Red White Dual Purpose, Norwegian Red, Swedish Red, Ayrshire Finnish, Hereford, Angus, Normande, Montbeliarde, Shorthorn, Brown Swiss, Jersey, and Wagyu, but lower than in Modern Danish Red, Kholmogory, Charolais, Gelbvieh, Limousin, Simmental, Original Braunvieh, Fleckvieh, and Yakut (Supplementary Table S3). The values across all breeds for π_TotMean_ and H_o_ (*r* = 0.87, *p* = 4.5 × 10^−8^) as well as between π_TotMean_ and H_e_ (*r* = 0.88, *p* = 1.7 × 10^−8^) correlated highly significantly (Supplementary Table S5).

When looking at the nucleotide diversity per chromosome, π_ChrMean_ values in DSN ranged between 0.134% on BTA 22 and 0.192% on BTA 23. The highest nucleotide diversity was evident in all breeds on BTA 23 in the window between 19 and 37 Mb (Figure 3B; Supplementary Table S6). This highly polymorphic region contains many protein-coding genes including the major histocompatibility complex (MHC) and the bovine leukocyte antigen-BoLA. Consistent with the total genomic nucleotide diversity, Jersey and Yakut showed the lowest and highest levels of diversity for most of the chromosomes, respectively (Figure 3B).

### 3.5 Genomic inbreeding

The inbreeding rate estimated as excess of homozygosity (F_Hom_) ranged from 2.7% in DSN to 9.8% in Hereford (Figure 3C). Hence, F_Hom_ in DSN was lower than in Holstein (6.4%) and all the other breeds. As expected, commercial breeds generally showed higher inbreeding rates, as seen for example in Charolais (7.0%), Shorthorn (8.5%), Brown Swiss (9.4%), and Hereford (9.8%).

In total, 1,337,471 RoHs were detected in the 302 sequenced DSN animals with an average length of 102 kb ranging from 526 bp to 14 Mb. On average, 4,428 RoHs were detected per DSN animal. In 150 Holstein animals, in total 507,198 RoHs were found with an average length of 128 kb ranging from 851 bp to 21 Mb. The average number of RoHs per Holstein animal was 4,226. This shows the presence of longer RoHs in Holstein in comparison to DSN.

The inbreeding rate as estimated by F_RoH_ and considering all RoHs longer than 50 kb was on average 16.0% in DSN and 19.7% in Holstein (Figure 3C). From F_RoH>50kb_ to F_RoH>2Mb_, lowest and highest inbreeding was always observed in Modern Danish Red and Jersey, respectively, while F_RoH>4Mb_ ranged from 0.7% in Limousin to 4.0% in Brown Swiss (Figure 3C). F_RoH>1Mb_ and F_RoH>2Mb_ showed similar results to those detected in F_Hom_ (Figure 3C). Significant Pearson’s correlations of 0.60 (*p* = 1.9 × 10^−3^) and 0.62 (*p* = 1.3 × 10^−3^) were found between F_Hom_ and F_RoH>1Mb_, and F_Hom_ and F_RoH>2Mb_, respectively (Supplementary Table S5).

### 3.6 Region-wide F_ST_ between DSN and Holstein

**FIGURE 4:**
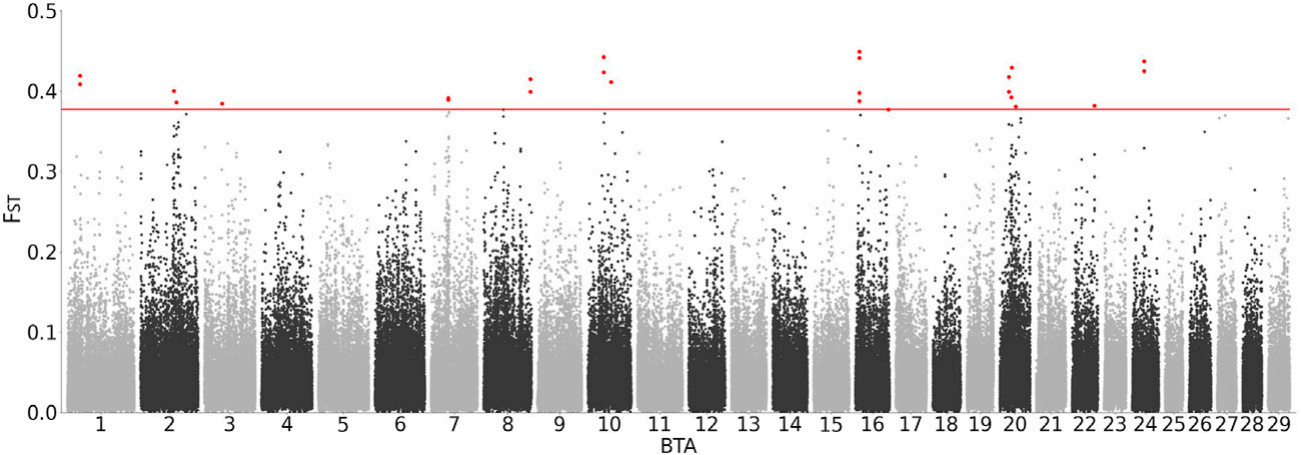
Region-wide F_ST_ between DSN and Holstein. All values were plotted in windows of 10 kb.

The analysis of F_ST_ values as a measure for the genomic diversity between DSN and Holstein revealed regions of high differentiation between those populations (Figure 4). Peaks of high F_ST_ values for windows of 10 kb length were identified on BTA 1, 2, 3, 7, 8, 10, 16, 20, 22 and 24. F_ST_ values of 25 10 kb-regions were above the threshold of 0.38 which represents the 0.01 percentile of the F_ST_ distribution. Since the average F_ST_ between DSN and Holstein was 0.069, most variants showed only low F_ST_ values.

The genes encoding 1,4-alpha-glucan branching enzyme 1 (*GBE1*), HECT, C2 and WW domain containing E3 ubiquitin protein ligase 2 (*HECW2*), and doublecortin domain containing 2C (*DCDC2C*) are located inside the 10 kb-regions on BTA 1, 2, and 8, respectively, with F_ST_ values between DSN and Holstein above the threshold. Additional 71 genes are located 250 kb up- or downstream of the start and end positions of the 25 detected 10 kb-regions above the threshold (Table 2). Regions on BTA 16 and 24 do not contain protein-coding genes. Annotation on QTLs and associations of all regions from CattleQTLdb are shown in Supplementary Table S7.

**TABLE 2:** Positional candidate genes in regions with the highest F_ST_ values between DSN and Holstein. Genes in bold are located directly inside the windows with the highest F_ST_ values. All other genes are located ± 250 kb from the start and end positions of the windows with the highest F_ST_ values. Consecutive windows displaying the same genes are shown together, this is the case for windows in BTA 1, 7, 8, and 10, whereof length is 20 kb.

Location	SNP count	Length (kb)	Genes ± 250 kb
1 : 29,280,001–29,300,000	279	20	1,4-alpha-glucan branching enzyme 1 (***GBE1***), *ENSBTAG00000008359*
2 : 78,690,001–78,700,000	91	10	Glycophorin C (*GYPC*)
2 : 84,980,001–84,990,000	88	10	HECT, C2 and WW domain containing E3 ubiquitin protein ligase 2 (***HECW2***)*,* coiled-coil domain containing 150 (*CCDC150*)
3 : 41,600,001–41,610,000	97	10	Olfactomedin 3 (*OLFM3*)*, ENSBTAG00000051863*
7 : 41,060,001–41,080,000	346	20	Germinal center associated signaling and motility like (*GCSAML*), olfactory receptor family 2 subfamily C member 3 (*OR2C3*), and 3B (*OR2C3B*)*,* subfamily G member 2 (*OR2G2*), 3 (*OR2G3*), and 27 *(OR2G27*)*,* subfamily W member 3 *(OR2W3*), and 3D (*OR2W3D*)*,* subfamily AO member 1 (*OR2AO1*)*,* subfamily T member 54 (*OR2T54*), family 5 subfamily AE member 3 (*OR5AE3*), and 4 (*OR5AE4*)*,* family 6 subfamily F member 1 (*OR6F1*)*,* subfamily AA member 1 (*OR6AA1*)*,* subfamily AN member 1 (*OR6AN1*)*,* family 9 subfamily E member 2 (*OR9E2),* family 11 subfamily L member 1 (*OR11L1*)*,* family 14 subfamily P member 2 (*OR14P2*)*,* tripartite motif containing 58 (*TRIM58*)*, ENSBTAG00000030735*
8 : 110,900,001–110,920,000	265	20	Doublecortin domain containing 2C (***DCDC2C***)*,* gelsolin (*GSN*)*,* stomatin (*STOM*)*,* allantoicase (*ALLC*)*,* collectin subfamily member 11 (*COLEC11*)*,* ribosomal protein S7 (*RPS7*)*,* ribonuclease H1 (*RNASEH1*)*,* acireductone dioxygenase 1 (*ADI1*), trafficking protein particle complex subunit 12 (*TRAPPC12*)*,* EARP complex and GARP complex interacting protein 1 (*EIPR1*)*, ENSBTAG00000049154*
10 : 38,480,001–38,500,000	206	20	Ubiquitin protein ligase E3 component n-recognin 1 (*UBR1*)*,* transmembrane protein 62 (*TMEM62*)*,* cyclin D1 binding protein 1 (*CCNDBP1*)*,* erythrocyte membrane protein band 4.2 (*EPB42*)*, ENSBTAG00000046363*
10 : 55,600,001–55,610,000	177	10	Unc-13 homolog C (*UNC13C*)
20 : 27,500,001–27,510,000	77	10	ISL LIM homeobox 1 (*ISL1*)
20 : 28,610,001–28,620,000	119	10	Poly (ADP-ribose) polymerase family member 8 (*PARP8*)*,* embigin (*EMB*)
20 : 37,390,001–37,400,000	101	10	Ciliosis and planar polarity effector 1 (*CPLANE1*)*,* NIPBL cohesin loading factor (*NIPBL*)*, ENSBTAG00000050782,* solute carrier family 1 member 3 (*SLC1A3*)
22 : 52,820,001–52,830,000	105	10	Coiled-coil domain containing 12 (*CCDC12),* parathyroid hormone 1 receptor (*PTH1R*)*,* myosin light chain 3 (*MYL3*)*,* serine protease 42 (*PRSS42P*)*, ENSBTAG00000037821, ENSBTAG00000052304, ENSBTAG00000049544, ENSBTAG00000050911,* serine protease 45 (*PRSS45*)*, ENSBTAG00000038616, ENSBTAG00000005019,* transmembrane inner ear (*TMIE*)*,* ALS2 C-terminal like (*ALS2CL),* leucine rich repeat containing 2 (*LRRC2*)*,* teratocarcinoma-derived growth factor 1 (*TDGF1*)*,* receptor transporter protein 3 (*RTP3*)*,* lactotransferrin (*LTF*)*,* C-C motif chemokine receptor like 2 (*CCRL2*)*,* receptor 5 (*CCR5*), and 2 (*CCR2*)

### 3.7 Cross-population-extended haplotype homozygosity

**FIGURE 5:**
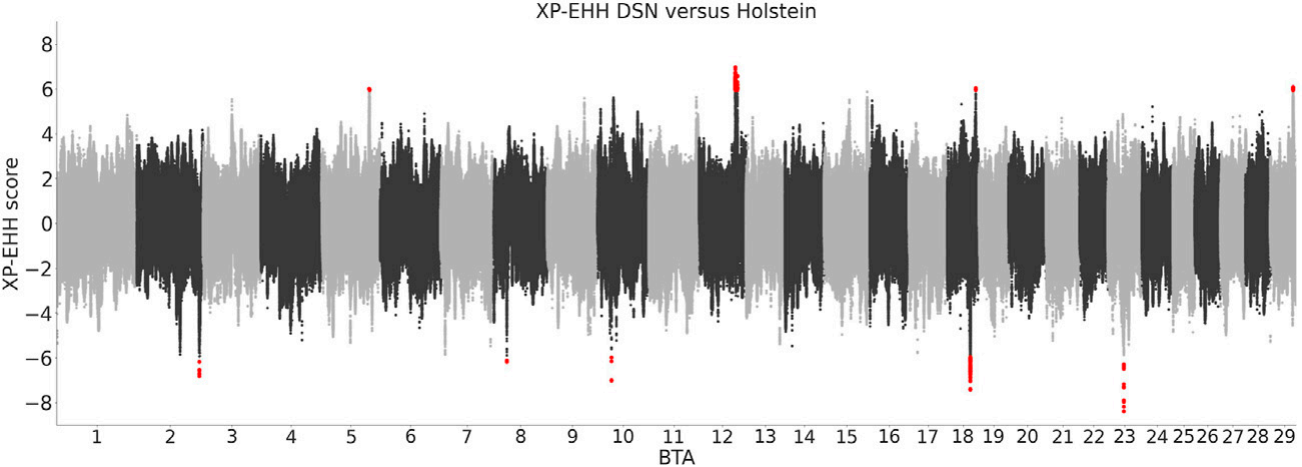
XP-EHH scores between DSN and Holstein. Positive scores represent variants positively selected in DSN in comparison to Holstein, and negative scores represent variants positively selected in Holstein in comparison to DSN. Significant scores are shown in red.

The analysis of XP-EHH identified 140 variants positively selected in DSN in comparison to Holstein and 80 variants positively selected in Holstein in comparison to DSN (Figure 5). Those variants are located in DSN in four regions on BTA 5, 12, 18, and 29, and in Holstein in five regions on BTA 2, 8, 10, 18, and 23 (Table 3). The highest number of significant variants was detected on BTA 12 in DSN (124 variants) and on BTA 18 in Holstein (57 variants). 73% of the significant variants were intronic, while 26% were intergenic and 1% downstream of genes.

**TABLE 3:** Positional candidate genes in regions with significant XP-EHH regions between DSN and Holstein. Genes in bold are located directly within the regions. All other genes are located ± 250 kb from the start and end positions of the regions.

Location	Length (kb)	SNP count	Breed whereof positively selected	Genes ± 250 kb
2 : 130,479,391–130,479,547	0.156	6	Holstein	EPH receptor A8 (*EPHA8*), Zinc finger and BTB domain containing 40 (*ZBTB40*), Wnt family member 4 (*WNT4*)
8 : 23,020,644–23,020,649	0.005	2	Holstein	*ENSBTAG00000048891*, Interferon-tau-like (*IFN-TAU*), *ENSBTAG00000054099, ENSBTAG00000055152*, Kelch like family member 9 (*KLHL9*), Interferon alpha G (*IFNAG*), *ENSBTAG00000050194, ENSBTAG00000052859, ENSBTAG00000048428, ENSBTAG00000051881, ENSBTAG00000053037, ENSBTAG00000053413*, Interferon beta 3 (*IFNB3*), *ENSBTAG00000046967*
10 : 23,771,505–23,771,788	0.283	4	Holstein	*ENSBTAG00000051554, ENSBTAG00000048374, ENSBTAG00000052580,* ENSBTAG00000048874, T cell receptor alpha variable 24 (*TRAV24*), *ENSBTAG00000052314*
18 : 51,426,960–51,445,790	18.830	57	Holstein	*ENSBTAG00000054584*, Glutamate ionotropic receptor kainate type subunit 5 (*GRIK5*), ATPase Na+/K+ transporting subunit alpha 3 (*ATP1A3*), Rab acceptor 1 (*RABAC1*), *ENSBTAG00000053222*, Rho guanine nucleotide exchange factor 1 (*ARHGEF1*), CD79a molecule (*CD79A*), Ribosomal protein S19 *(RPS19*), DMRT like family C2 (*DMRTC2*), LY6/PLAUR domain containing 4 (*LYPD4*), *ENSBTAG00000006859,* ***ENSBTAG00000049346*** *, ENSBTAG00000052343, ENSBTAG00000054156*, C-X-C motif chemokine ligand 17 (*CXCL17*), CD177 molecule (*CD177*), Testis expressed 101 (*TEX101*), Binder of sperm 1 (*BSP1*), 3 (*BSP3*), and 5 (*BSP5*), *ENSBTAG00000049614*, LY6/PLAUR domain containing 3 (*LYPD3*), Pleckstrin homology like domain family B member 3 (*PHLDB3*)
23 : 26,112,509–26,271,305	158.796	11	Holstein	Butyrophilin like 2 (*BTNL2*), *ENSBTAG00000034945, ENSBTAG00000007618,* ***ENSBTAG00000026163, ENSBTAG00000050817***
5 : 99,140,148–99,322,102	181.954	4	DSN	Serine/threonine/tyrosine kinase 1 (*STYK1*), Mago homolog B exon junction complex subunit (*MAGOHB*), *ENSBTAG00000009252, ENSBTAG00000052865, ENSBTAG00000046268,* ***ENSBTAG00000049367*** *,* ***ENSBTAG00000054018, ENSBTAG00000054633*** *, ENSBTAG00000052486, ENSBTAG00000052514, ENSBTAG00000050324, ENSBTAG00000022861, ENSBTAG00000051183, ENSBTAG00000049823, ENSBTAG00000052658, ENSBTAG00000038843*
12 : 69,770,246–71,722,914	1,952.668	124	DSN	Multidrug resistance-associated protein 4 (*LOC515333*)*,* ***ENSBTAG00000047383, ENSBTAG00000046041, ENSBTAG00000049836***
18 : 61,391,310–61,398,262	6.952	3	DSN	*ENSBTAG00000000336, ENSBTAG00000009171, ENSBTAG00000015061, ENSBTAG00000014328, ENSBTAG00000054918, ENSBTAG00000013345, ENSBTAG00000009364, ENSBTAG00000015987, ENSBTAG00000051856, ENSBTAG00000046961, ENSBTAG00000051149, ENSBTAG00000030416, ENSBTAG00000015139, ENSBTAG00000018152*, Protein kinase C gamma (*PRKCG*), Calcium voltage-gated channel auxiliary subunit gamma 7 (*CACNG7*), and 8 (*CACNG8*)
29 : 38,542,360–38,548,547	6.187	9	DSN	*ENSBTAG00000051614, ENSBTAG00000039970, ENSBTAG00000050440, ENSBTAG00000040340*, Pregnancy-associated glycoprotein 1 (*PAG1*), *ENSBTAG00000048202, ENSBTAG00000054803, ENSBTAG00000051196*

Seven genes are located directly within the identified XP-EHH regions, among them four in DSN and three in Holstein. When considering genes located 250 kb up- or downstream of the start and end positions of the regions, 110 genes are detected, 49 in DSN, and 61 in Holstein. Furthermore, no overlaps were detected between regions with high F_ST_ and XP-EHH regions. Annotation of genes in QTLs and associations of all regions from CattleQTLdb are shown in Supplementary Table S8.

### 3.8 RoH islands

Based on the frequency of SNPs inside RoHs, 21 RoH islands were detected in DSN and 19 in Holstein. Supplementary Figure S5 shows the frequency of SNPs inside RoHs for DSN and Holstein, and the threshold used in each case to define islands (0.66 in DSN and 0.61 in Holstein). Regions on BTA 1, 4, 14, and 18, where RoH islands were detected in both breeds in the same location, likely contributed essentially to selection (Table 4).

**TABLE 4:** Location of RoH islands and genes inside RoH islands in DSN and in Holstein. Genes in bold are common between breeds.

BTA	RoH island in DSN		RoH island in Holstein	
	Location	Genes	Location	Genes
4	76,848,259–76,993,327	Transmembrane p24 trafficking protein 4 (***TMED4),*** DEAD-box helicase 56 (***DDX56***), NPC1 like intracellular cholesterol transporter 1 (***NPC1L1***), NudC domain containing 3 (***NUDCD3***)	76,847,893–76,984,479	Transmembrane p24 trafficking protein 4 (***TMED4),*** DEAD-box helicase 56 (***DDX56***), NPC1 like intracellular cholesterol transporter 1 (***NPC1L1***), NudC domain containing 3 (***NUDCD3***)
6	35,779,310–35,831,557	Family with sequence similarity 13 member A (*FAM13A*)		
8			106,068,712–106,290,180 (with gaps, see Supplementary Table S9)	Astrotactin 2 (*ASTN2*)
10	30,721,933–30,889,605	Diphthamine biosynthesis 6 (*DPH6*)		
13			437,055–501,916 (with gaps, see Supplementary Table S9)	*ENSBTAG00000024139, ENSBTAG00000051498,* olfactory receptor family 4 subfamily C member 27 (*OR4C27*)
14	22,768,759–23,297,958 (with gaps, see Supplementary Table S9)	XK related 4 (***XKR4***), transmembrane protein 68 (***TMEM68***), trimethylguanosine synthase 1 (***TGS1),*** LYN proto-onco, Src family tyrosine kinase (***LYN)*** *,* ribosomal protein S20 (*RPS20*)	22,768,535–23,225,387 (with gaps, see Supplementary Table S9)	XK related 4 (***XKR4***), transmembrane protein 68 (***TMEM68***), trimethylguanosine synthase 1 (***TGS1),*** LYN proto-onco, Src family tyrosine kinase (***LYN)***
	31,316,339–31,538,677 (with gaps, see Supplementary Table S9)	Centrosome and spindle pole associated protein 1 (*CSPP1*)*,* ADP ribosylation factor guanine nucleotide exchange factor 1 (*ARFGEF1*)*,* Carboxypeptidase A6 (*CPA6*)		
16	40,659,742–41,948,518 (with gaps, see Supplementary Table S9)	TNF superfamily member 18 (*TNFSF18*), mitofusin 2 (*MFN2*), procollagen-lysine,2-oxoglutarate 5-dioxygenase 1 (*PLOD1*), angiotensin II receptor associated protein (*AGTRAP*)		
17	60,987,746–61,013,323	LIM homeobox 5 (*LHX5*), serine dehydratase like (*SDSL*)		
18	14,422,668–14,490,133	Ankyrin repeat domain 11 (*ANKRD11*), SPG7 matrix AAA peptidase subunit, paraplegin (***SPG7***)	14,482,261–14,525,853	SPG7 matrix AAA peptidase subunit, paraplegin (***SPG7***), ribosomal protein L13 (*RPL13*), copine 7 (*CPNE7*), dipeptidase 1 (*DPEP1*)
	57,506,154–57,570,852 (with gaps, see Supplementary Table S9)	Zinc finger protein 175 (*ZNF175*), *ENSBTAG00000023365, ENSBTAG00000045880*		
26			9,558,523–9,638,107	Phosphatase and tensin homolog (*PTEN*)

In total, 26 genes were found inside all RoH islands in DSN. For Holstein, 17 genes were located inside all RoH islands. Between DSN and Holstein, nine genes overlapped with the RoH islands on BTA 4, 14, and 18. Genes occurring 250 kb up- or downstream from start and end positions of RoH islands are shown in Supplementary Table S9.

No exact overlap was detected between RoH islands and regions with high F_ST_ or XP-EHH regions, neither in DSN nor in Holstein. In DSN, the smallest distance of a RoH island to an XP-EHH region was 3.8 Mb on BTA 18 (*R* ^2^ = 0.001, D’ = 0.12), and to an F_ST_ window 7.5 Mb on BTA 10 (*R* ^2^ = 0.001, D’ = 1). In Holstein, the smallest distance of a RoH island to an XP-EHH region was 3.0 Mb on BTA 20 (*R* ^2^ = 0.03, D’ = 0.22) and to an F_ST_ window 4.6 Mb on BTA 8 (*R* ^2^ = 0.03, D’ = 0.23).

## 4 Discussion

### 4.1 Relationship analyses

Considering the origin of DSN in the North Sea region, our expectation of cattle breeds from the same region such as Dutch Friesian Red, Holstein, and other breeds share a common ancestry with DSN was confirmed. But interestingly, Holstein was not the closest related breed to DSN, neither in F_ST_ nor phylogenetic analyses, despite their shared history. Asian and Eastern European countries, and Central Italy showed the closest cluster to Auroch, indicating the most ancient origin. Cattle domestication in Europe is believed to have started in Italy, with further migration of those cattle to Central and Northern Europe (Felius et al., 2014), which is consistent with the clusters of breeds in our phylogenetic tree. In addition, our clusters are also consistent with clusters previously reported by other genetic studies (Felius et al., 2011) that are also based on WGS data of cattle (Dutta et al., 2020). Since pruning parameters slightly affect the tree construction, and considering the complexity of the development of the cattle breeds in Europe (Felius et al., 2014), we cannot entirely explain how much the true relationship between breeds differs from our or reported findings.

We identified an F_ST_ value of 0.069 between DSN and Holstein. This value is consistent with the estimate of 0.068 which has been reported using 261 and 4,654 animals from Illumina BovineSNP50 Beadchip data, respectively (Naderi et al., 2020). Generally, F_ST_ values support our phylogenetic findings. Nevertheless, small differences were seen in the order of closest related breeds to DSN based on F_ST_ values, and breeds inside clusters closely related to DSN in the phylogenetic tree. This may be caused by the fact that the UPGMA algorithm used the average distance between all investigated animals at once to build the phylogenetic tree, while F_ST_ values were calculated pairwise between DSN and each breed separately. The latter might slightly bias the analysis towards those two breeds. In addition, the data used for the phylogenetic tree was pruned, while F_ST_ values were calculated using only unpruned variants segregating between two investigated breeds.

Although Charolais was not located inside the Northern European cluster in the phylogenetic tree, it showed a low F_ST_ value of 0.070 with DSN. Such a low F_ST_ value of 0.074 was also reported between Charolais and Holstein (Kelleher et al., 2017). The admixture and f3 analyses provided evidence for some admixture between Charolais and some other breeds from Northern Europe. Additionally, a migration event was observed between an ancestor of two breeds from the Northern European cluster, Shorthorn and Maine Anjou, to Charolais, which might explain the admixture results. An influence of shorthorn on Charolais –or of Durham cattle, ancestor of Shorthorn and Maine Anjou – has been reported before from the breeding history. The two breeds were separated by establishing independent herd books only in 1890 (Felius et al., 2014).

High admixture was observed between Modern Angler, Modern Danish Red, German Red Angler, and Norwegian Red, causing wrong assignments between those breeds in our phylogenetic analysis. This admixture, however, is consistent with recent findings (Schmidtmann et al., 2021).

Finally, we have to mention that every relationship study is dependent on the number of animals per breed, their kinship, and how good the given animals represent the breed. In our study, the number of animals per breed varied from 5 to 302, for diversity analyses from 28 to 302 sticking to FAO guidelines (FAO, 2011). We had no information on the relatedness of individuals and how good the animals represent each breed.

### 4.2 Genomic diversity

The average total nucleotide diversity (π_TotMean_) of DSN was similar to other breeds from Northern Europe, but slightly above average indicating good population management. Eastern European and Asian breeds showed higher π_TotMean_ values than Northern and Central European breeds, likely due to less intensive breeding programs, genetic bottlenecks, or founder effects in comparison to the other breeds (Felius et al., 2014). The highest average chromosomal nucleotide diversity (π_ChrMean_), which was found on BTA 23 at 19–37 Mb, can be attributed to the highly polymorphic region of the MHC. This high diversity around the MHC is observed in all mammals (Shiina et al., 2017).

Even though there was a high correlation between π_TotMean_ and H_o_ and H_e_, DSN showed the lowest H_e_ and one of the lowest H_o_ in comparison to other breeds, differently from its π_TotMean_ result. One reason for this discrepancy might be the large number of 302 DSN animals in comparison to other breeds. However, this hypothesis was discarded since no correlation was detected between the number of animals per breed and π_TotMean_. Furthermore, π_TotMean_ calculated for either 50 (0.1513%) or 302 DSN animals (0.1506%) which was almost identical. Nevertheless, no statistical difference was detected between H_o_ and H_e_ for DSN. The high nucleotide diversity and low heterozygosity likely results from the small herd size of DSN with 2,452 cows and only 36 breeding bulls (TGRDEU, 2021).

The breeds Jersey and Yakut showed the lowest and highest levels of diversity, respectively. Jersey cattle, which were originally restricted to the Jersey island, suffer from high inbreeding and low genetic diversity rates, which is seen even among animals not kept on the islands anymore (Huson et al., 2020). Lower genetic diversity is expected for insular than for continental populations (Frankham, 1997). Yakut, in contrast, is an ancient breed from Siberia, known for its adaptation to extreme low temperatures. The reported high nucleotide diversity of 0.173% in this breed might be due to lower artificial selection and a higher effective population size of the ancestral Asian taurine in comparison to European cattle (Weldenegodguad et al., 2019).

### 4.3 Genomic inbreeding

Inbreeding measured by the homozygosity index F_Hom_ was generally higher for intensively selected breeds such as Hereford, Brown Swiss, Shorthorn, Charolais, and Holstein. Jersey also showed particular high inbreeding. DSN had the lowest excess of homozygosity index, which demonstrates its good management as a genetic resource. This shows that in a local breed with only about 2,500 animals, diversity can be maintained through a breeding scheme that aims at less intensive selection for production traits (Gutiérrez-Reinoso et al., 2022), which is strongly different from Holstein, for example, where millions of animals are kept. In our analysis, Holstein cattle were available from four countries, the United States, Denmark, Germany, and the Netherlands, which probably led to slightly higher diversity rates than having only Holsteins from one country.

The same pattern for inbreeding was observed when using F_RoH_ as indicated by the high correlation rates between inbreeding metrices. High correlation between F_Hom_ and F_RoH_ was also observed in the literature (Mastrangelo et al., 2016). Our F_RoH_ values were consistent with those previously reported, for example, for Original Braunvieh (F_RoH>50 kb_ = 14.58%) (Bhati et al., 2020), Modern Danish Red (F_RoH>10 kb_ = 11.84%), Holstein (F_RoH>10 kb_ = 18.67%), and Jersey (F_RoH>10 kb_ = 24.23%) (Zhang et al., 2015). Another aspect is the length of regions of homozygosity which reflects recent and ancient inbreeding. Longer RoHs indicate more recent inbreeding, while shorter RoHs indicate ancient inbreeding (Makanjuola et al., 2020). Moreover, recent inbreeding shows higher detrimental inbreeding depression effects (Makanjuola et al., 2020). Commercial breeds show more often long RoHs (Marras et al., 2015). This was the case for Hereford and Brown Swiss. Nevertheless, non-commercial breeds such as Jersey, Wagyu, and even Yakut also showed high average RoH lengths and high F_RoH>4 Mb_ (Supplementary Table S4). DSN showed fewer longer RoHs, which were generally shorter, than in other breeds.

### 4.4 Signatures of selection

Signatures of selection were particularly examined in DSN and Holstein cattle. Considering DSN as a dual-purpose breed for milk and meat production, and Holstein as a high-yielding dairy breed, differentiated regions are expected to contain genes associated with traits influencing meat production, carcass, body conformation, and milk production.

#### 4.4.1 Region-wide F_ST_ between DSN and Holstein

In total, we identified 25 high differentiating F_ST_ windows likely reflecting signatures of selection in DSN when comparing to Holstein. Two regions on BTA 20 and 10 have been detected previously (Naderi et al., 2020). All other regions were novel. Inside the top high differentiating F_ST_ regions, the three genes *GBE1*, *HECW2*, *DCDC2C* were located*. GBE1* on BTA 1 was described in literature as responsible for the production of the glycogen branching enzyme, therefore, being involved in the carbohydrate and glycogen metabolisms. Glycogen is an important short-term energy storage molecule in the muscle. *GBE1* was also detected in signatures of selection using RoHs in U.S. Holstein cattle (Kim et al., 2013). Its deficiency has also been associated with glycogen storage diseases and stillbirths in humans, cattle, and equines (Ward et al., 2004; Lee et al., 2011; Lorenz et al., 2011; Almodóvar-Payá et al., 2020). Furthermore, the F_ST_ region where *GBE1* was located overlapped with two QTLs from CattleQTLdb for production traits (Crispim et al., 2015; Hamidi Hay and Roberts, 2017), growth and longevity (Supplementary Table S7). *HECW2* on BTA 2 is responsible for protein ubiquitination. The gene was described as a candidate gene for milking speed in French Holstein (Marete A. et al., 2018b), aging and angiogenesis in humans (Rotin and Kumar, 2009; Walter et al., 2011; Choi et al., 2016; Berko et al., 2017). *DCDC2C* on BTA 8 was suggested to be associated with sperm formation in cattle (Wu et al., 2020), structural defects in cilia in sperm (Jumeau et al., 2017) and in cilia length in sensorial cells in humans (Grati et al., 2015). Moreover, the same region on BTA 8 presented a QTL for meat in the CattleQTLdb (McClure et al., 2012). Those genes point for the differentiation between signatures of selection between DSN and Holstein, including candidates for meat, milk, production, and fertility traits.

In addition to genes located directly inside the topmost differentiated genomic regions between DSN and Holstein, genes within 250 kb up- or downstream were analyzed. *GYPC* on BTA 2 has been previously reported as a candidate gene for body length (Vanvanhossou et al., 2020) and subclinical ketosis in Holstein (Soares et al., 2021), while *CCDC150* was reported as candidate for milk and fat yield in Nordic Holstein cattle (Cai et al., 2020). BTA 7 showed a series of olfactory receptor genes. *GCSAML,* a germinal center associated signaling and motility like gene of mature B lymphocytes, was reported to be significantly down-regulated in Holstein cows under heat-stress conditions (Kim et al., 2021). Mammalian olfactory receptors are encoded by the largest mammalian multigene family, containing 881 genes on 26 chromosomes. Studies suggest physiological and behavior aspects of variation of olfactory receptor genes, e.g., associated to appetite regulation in livestock (Connor et al., 2018), which influences uptake of nutrients required for milk and meat production. This corroborates the idea of DSN well adapted to grazing with high roughage feed intake. In the BTA 7 region, QTLs for milk and meat production were found in the CattleQTLdb (Daetwyler et al., 2008; McClure et al., 2010; Marete A. et al., 2018b).

Other interesting genes near topmost differentiated genomic regions between DSN and Holstein are *CCNDBP1* on BTA 10, a candidate for skeletal myogenesis (Huang et al., 2016); *UNC13C* on BTA 10 a candidate for feed efficiency (Freua et al., 2016); *EMB* on BTA 20 a candidate for mammary gland tissue development (Butty et al., 2017); *NIPBL* on BTA 20 a candidate for growth (de Simoni Gouveia et al., 2017; Wang et al., 2022) and previously detected as positively selected in German Holstein in the study between DSN and German Holstein using Illumina BovineSNP50 Beadchip (Naderi et al., 2020); and *ALS2CL*, *LRRC2*, and *TDGF1* on BTA 22, which are candidates for milk production (Ibeagha-Awemu et al., 2016) and fertility (Wei et al., 2017; Tríbulo et al., 2018), and previously detected in highly differentiated regions between recent populations of Dutch Frisian and Holstein (Hulsegge et al., 2022). In the region on BTA 22, the gene *LTF* (lactotransferrin) was found which is a major iron-binding protein in milk and body secretions of bovine (Rejman et al., 1989; Pierce et al., 1991) with an antimicrobial activity (Bellamy et al., 1992). Furthermore, *LTF* was reported to influence casein yield (Cecchinato et al., 2014). Although the region on BTA 24 did not contain any gene, the same region was associated with endoparasite resistance in DSN (May et al., 2019).

#### 4.4.2 Cross-population-extended haplotype homozygosity

Out of the four regions positively under selection in DSN, two are novel and two had been previously reported (Naderi et al., 2020). The region on BTA 12 has been reported before as positively selected in DSN in a study between DSN and German Holstein using Illumina BovineSNP50 Beadchip (Naderi et al., 2020). In this region, *LOC515333* resides, a novel gene that has been so far annotated as coding the multidrug resistance-associated protein 4 (NCBI gene ID 515333). In cattle, this protein was reported to influence fertility traits since it is involved in the transport of prostaglandins and the regulation of oxytocin (Lacroix-Pépin et al., 2011). The XP-EHH region on BTA 18 which is close to the RoH island on the same chromosome also corroborates previous findings (Naderi et al., 2020). This region contains for instance *CACNG7,* a gene that was reported as a candidate gene for feed efficiency in Nellore cattle (Olivieri et al., 2016).

The regions on BTA 5 and 29 are novel. The region on BTA 5 has been repeatedly associated with milk production (Olsen et al., 2002; Bennewitz et al., 2003; Meredith et al., 2012; Rutten et al., 2013; Jiang et al., 2019). This region contains, for example, the gene *STYK1*, which has been reported as a candidate gene for heat stress response demonstrated through milk fatty acids alterations in German Holstein (Bohlouli et al., 2022). The region on BTA 29 contains the well described gene *PGA1* (Xie et al., 1995; Klisch et al., 2005; López-Gatius et al., 2007), encoding the pregnancy associated glycoprotein-1, which is expressed in the placenta where it is crucial for a healthy gestation in cattle.

For Holstein, regions on 5 chromosomes were found. Only, the region on BTA 2 corroborates the previous study comparing DSN and German Holstein (Naderi et al., 2020). This is likely due to the different Holstein populations used in the different studies. Naderi *et al.* used Holstein cattle from Germany, while the current study used Holstein cattle from the 1000 Bull Genomes project (Hayes and Daetwyler, 2019) which originate from four different countries. The selection region on BTA 2, for instance, contains the *WNT4* gene, which affects ovulation (Tríbulo et al., 2018), neurogenesis and embryogenesis in cattle (Gao et al., 2016).

The four other selection regions detected in Holstein contain genes such as *IFNB3* on BTA 8, reported with evidence of inhibition against the bovine herspesvirus type 1 (da Silva et al., 2012), *TRAV24* on BTA 10 which is T-cell receptor, *BSP1*, *BSP2*, and *BSP3* on BTA 18 which bind the sperm proteins 1,3 and 5, respectively (D’Amours et al., 2012), and *BTNL2* on BTA 23, which encodes butyrophilin (Afrache et al., 2012), as part of the immunoglobulin superfamily of transmembrane proteins in the MHC.

The absence of an overlap between region-wide F_ST_ and XP-EHH analyses is due to differences in the two methods, which are complementary. F_ST_ values were calculated based on allele frequencies, while XP-EHH values were calculated based on the decay of haplotype homozygosity, reflecting more recent selection signatures (Sabeti et al., 2007).

#### 4.4.3 RoH islands

When signatures of selection were analyzed within breeds, nine genes were detected by RoH islands in both DSN and Holstein. Those nine genes are located on BTA 4, 14, and 18. The region on BTA 4 has been reported for mastitis resistance (Rupp and Boichard, 2003), growth (Lu et al., 2013), fertility (Grigoletto et al., 2020b), and primarly milk (Ibeagha-Awemu et al., 2016; Sanchez et al., 2017, 2019; Van Den Berg et al., 2020), while the region on BTA 14 was primarly reported for meat (Bolormaa et al., 2011; Neto et al., 2012; Saatchi et al., 2014; Sharma et al., 2014; Ali et al., 2015; Song et al., 2016; Kim et al., 2017; Akanno et al., 2018; Grigoletto et al., 2020a; Srikanth et al., 2020; Wang et al., 2020; Rezende et al., 2021), production (Snelling et al., 2010; Lu et al., 2013; Martínez et al., 2014; Saatchi et al., 2014; Akanno et al., 2018; Zhong et al., 2019; Zhang et al., 2020), and exterior traits (Pryce et al., 2011; Pausch et al., 2016; Wu et al., 2016; Marete A. G. et al., 2018; Bouwman et al., 2018; Tribout et al., 2020). Within this region, for example, we find the gene *XKR4* associated with subcutaneous rump fat thickness and growth (Neto et al., 2012; Magalhã et al., 2016; An et al., 2019; Smith et al., 2019)*.* Lastly, the region on BTA 18 was reported as a candidate region for tuberculosis resistance (Ring et al., 2019), milk (Cole et al., 2011; Ibeagha-Awemu et al., 2016; Benedet et al., 2019), and fertility traits (Cole et al., 2011; Gaddis et al., 2016) including spermatogenesis-associated proteins 33 and 2L (*SPATA33* and *SPATA2L*) in the extended regions of 250 kb.

Regions detected in DSN, but not in Holstein, were very often associated with meat and carcass. Among the RoH islands detected in each breed 76.2% in DSN were associated with meat, but only 47.4% in Holstein. For milk, 95.2% of RoH islands in DSN and 78.9% of RoHs islands in Holstein were associated with milk production. Interestingly, the RoH region on BTA 28, which was found in DSN only, previously has been associated with milk production traits in a genome-wide association study in DSN (Korkuć et al., 2021).

Although differences were seen in signatures of selection, many signatures are shared between DSN and Holstein. This is consistent with the genetic relatedness and shared history of DSN and Holstein and the fact that both breeds had been selected for milk yield.

Considering all the results from signatures of selection, traits related to meat and milk showed the largest differences between DSN and Holstein, due to different selection goals. Genes affecting fertility, exterior, production, and health traits were also very frequent. Those findings are consistent with the characteristics of the breed-type and purposes described by the breeding organizations.

## 5 Conclusion

Despite the small population size of 2,500 animals, the DSN breed does not show any signs of loss of diversity or increased inbreeding compared to other taurine breeds. On the contrary, the inbreeding degree in DSN is even lower and the diversity higher than in Holstein. This is a remarkable result of the breeding strategy used for the maintenance of DSN as a genetic resource and shows the potential of maintaining small local populations while keeping diversity and controlling inbreeding. Our study provides the background for cattle breeds that are closely related to DSN and could, therefore, serve as an external gene pool to keep or even increase the diversity in DSN. Our analyses also provide evidence for high genomic diversity in breeds such as Yakut, Charolais, Kholmogory, and Modern Danish Red, while inbreeding was high in Jersey, Wagyu, Hereford, and Shorthorn, pointing to extra care needed for those breeds.

Moreover, specific genomic regions and positional candidate genes seem to be partially responsible for the DSN-specific characteristics. These include candidate genes previously identified in association studies with DSN, such as one region detected in DSN for endoparasite infection resistance, an important trait for pasture systems. In addition, these regions point to genes associated with traits that have not been studied yet in DSN, but in other breeds or species. Such regions are likely of particular interest for the conservation of DSN and the maintenance of its specific characteristics. Further studies are needed in order to elucidate the function of those regions and underlying causal sequence variants. Besides investigating milk and beef production, the study of new traits for disease resistance and resilience, such as heat stress, methane emissions or feed uptake capacity can further improve our understanding of the importance of DSN as a small local breed and as a genetic resource that contributes to conserve the whole genomic diversity of the species.

## Data Availability

Publicly available datasets were analyzed in this study. This data can be found here: https://www.ebi.ac.uk/ena/browser/view/PRJEB45822. Data from other breeds was provided within the frame of the 1000 Bull Genomes Project Consortium (Run 9).
